# Biomarker Profile Does Not Predict Weight Loss Success in Successful and Unsuccessful Diet-Reduced Obese Individuals: A Prospective Study

**DOI:** 10.1155/2013/804129

**Published:** 2013-05-14

**Authors:** Sarit Polsky, Lorraine Garratt Ogden, Paul Scown MacLean, Erin Danielle Giles, Carrie Brill, Holly Roxanna Wyatt

**Affiliations:** ^1^Anschutz Health and Wellness Center, 12348 E. Montview Boulevard, Campus Box C263, Aurora, CO 80045, USA; ^2^University of Colorado Anschutz Medical Campus, Division of Endocrinology, Metabolism and Diabetes, 12800 E. 19th Avenue, Campus Box F8305, Aurora, CO 80045, USA

## Abstract

*Background*. Individuals attempting weight reduction have varying success when participating in the same intervention. Identifying physiological factors associated with greater weight loss could improve outcomes. *Methods*. Sixty-one adults (BMI 27–30 kg/m^2^) participated in a 16-week group-based, cognitive-behavioral control weight loss program. Concentrations of 12 fasting hormones and cytokines related to adiposity, satiety/hunger, and inflammation were measured using the Milliplex human metabolic human panel before and after weight loss. Participants were grouped based on ≥8% (successful group, SG) or <8% weight loss (less successful group, LSG). *Results*. The SG had 46 subjects (75.4%), while the LSG had 15 (24.6%). There were no differences in baseline sex distribution, age, weight, BMI, and body composition between groups. In the SG, baseline to the 16-week levels decreased significantly for c-peptide (1,030 versus 891 pg/mL, *P* = 0.002), insulin (665 versus 541 pg/mL, *P* = 0.001), and leptin (0.83 versus 0.58 ng/mL/kg fat, P < 0.001). None of the baseline analytes predicted greater weight loss. *Conclusions*. Successful weight loss was associated with changes in adiposity (less fat mass) and unfavorable hunger signals. No baseline biomarker profile was associated with weight loss success. Behavioral factors may have outweighed physiological signals for determining successful weight loss. This trial is registered with Clinicaltrials.gov NCT00429650.

## 1. Introduction

Overweight and obesity affect billions of people worldwide. There are many proven methods to lose weight, including lifestyle modification, pharmacotherapies, and bariatric surgery. Still, many people who attempt weight loss are not successful and of the ones who are most of them regain the lost weight [1, 2]. One thing that would help in developing more effective weight loss strategies would be a better understanding of the characteristics that predict the ability to lose and maintain weight.

Some studies have examined potential hormonal predictors of weight loss. Adipokines, such as leptin and adiponectin, have shown promise. Lower leptin at baseline has been shown to predict better acute weight loss [3] and better long-term weight loss maintenance [4] in dietary interventions. Higher baseline adiponectin has been associated with more weight loss [5]. Signals related to hunger and satiation have also been explored. Ghrelin has been implicated in weight loss maintenance, but only for men [6]. There has been much speculation about how these hormones may affect weight loss and weight regain, such as changes in fat oxidation and compensatory changes in appetite [3–6], but definitive answers remain elusive. Moreover, most of the previous work has focused on one to three hormones at a time; thus, it is unclear if a panel of hormones could be used to predict weight loss.

In this study, we evaluated biological predictors of weight loss success in a group of subjects enrolled in a weight management program that provided individualized goals for diet and physical activity. Fasting levels of 12 adiposity, hunger/satiety, inflammatory hormones, and cytokines were measured to determine if levels of these easily obtained clinical markers, either individually or as a combined biomarker profile, could predict weight loss success.

## 2. Methods

### 2.1. Study Participants

Overweight and obese subjects were recruited in cohorts of 4–10 participants from primary care physician referrals, newspaper notices, and public service announcements. Inclusion criteria were ages from 19 to 45 years, body mass index (BMI) between 27 and 40 kg/m^2^, and weight stability. Exclusion criteria included history of cardiovascular or other major disease, current smoking, and use of certain medications affecting weight. After screening for the inclusion/exclusion criteria, subjects underwent a behavioral evaluation and a history and physical exam by their family practice physicians or the study physician. The protocol for the study Long-term Study of Exercise in the Treatment of Obesity (LoSE IT) was approved by the Colorado Multiple Institutional Review Board at the University of Colorado Anschutz Medical Campus. The study was conducted in accordance with the Declaration of Helsinki. This ongoing prospective study is designed to evaluate the efficacy of weight loss maintenance interventions that differ by physical activity levels. All participants provided written informed consent.

### 2.2. Study Protocol

Subjects participated in a 16-week intervention for weight loss. Baseline resting metabolic rate (RMR) and physical activity levels were used to customize caloric deficit goals of 500 to 1000 kcal/day. Participants received a comprehensive, group-based, cognitive-behavioral weight control program (Colorado Weigh) developed at the University of Colorado. Colorado Weigh consisted of weekly group meetings led by registered dietitians following a behavioral curriculum aimed at lifestyle modification. Energy intake was adjusted as needed based on weekly weights prior to each meeting to obtain a 1-2 lb weight loss per week. Food diaries and meal plans were provided. Subjects who lost less than the anticipated rate of weight loss had the option of receiving 4 meal replacements and were encouraged to eat a calorie-controlled frozen meal for a final meal daily. The goal was to lose at least 8% of starting body weight by the end of the intervention. After completing the weight loss intervention, subjects were weight stabilized, and study measurements were obtained between weeks 16 and 20 of the study. 

### 2.3. Study Measurements

#### 2.3.1. Anthropometrics

Body weight was measured with a calibrated digital scale to the nearest 0.2 pounds while subjects wore light clothing and no shoes. Pounds were converted to kilograms. Height was measured with a wall mounted stadiometer to the nearest 0.1 cm. Body mass index (BMI) was calculated as weight (kg)/height^2^ (m).

#### 2.3.2. Body Composition

Body composition was assessed at baseline by DXA (Hologic-Discovery W, Hologic, Inc., Bedford, MA, USA). Scanning was performed according to standard manufacturer's procedures with participants lying in the supine position.

#### 2.3.3. Biochemical Analyses

Blood samples were collected after an overnight fast at baseline and between weeks 16 and 20 of the study. Concentrations of 12 hormones and cytokines were simultaneously measured in plasma samples using the Milliplex human metabolic hormone panel (HMH-34 K, Millipore, St. Charles, MO, USA). Protease inhibitor cocktail (Sigma P2714) and AEBSF (Sigma A8456) were prepared according to the manufacturers' instructions and added to frozen plasma samples prior to thawing the plasma samples on ice. Analytes measured in this analysis included insulin, leptin, ghrelin, amylin, glucagon-like peptide-1(GLP-1), interleukin-6 (IL-6), tumor necrosis factor-alpha (TNF-*α*), monocyte chemotactic protein-1 (MCP-1), gastric inhibitory polypeptide (GIP), c-peptide, pancreatic polypeptide (PP), and peptide YY (PYY).

### 2.4. Data Analysis

Data analysis was performed with SAS Version 9.3 (SAS Institute, Inc., Cary, NC, USA).

Due to the positive skew observed in the distributions of the biomarkers, the data were log transformed for analysis. Leptin was adjusted for fat mass prior to transformation by dividing by kilograms of fat mass. Linear mixed effects models were estimated using restricted maximum likelihood in the SAS MIXED procedure to compare biomarker levels between those with ≥8% versus <8% weight loss. The correlation between repeated measures taken on the same individual was accounted for in the residual covariance structure using a symmetric covariance (i.e., assuming equal variances for pre- and post-weight loss). The covariance structure was also assumed to be equal in the two weight loss groups. This was considered the best covariance structure for the log-transformed data after examining the Akaike Information Criterion and Bayesian Information Criterion from all models. Model estimates were back-transformed and were reported as geometric means. Linear regression models were also fit regressing percent weight loss during the weight loss intervention (treated as a continuous outcome variable) on the natural log of each baseline biomarker and on change in the natural log of each biomarker. 

## 3. Results

### 3.1. Participant Characteristics

Sixty-five participants were enrolled in the study. Four participants withdrew from the study and were excluded from all analyses. Of the 61 remaining participants who completed the weight loss intervention, 46 (75.4%) achieved weight loss of ≥8% based on baseline weight. Fifteen participants (24.6%) lost <8% of their baseline weight. Nine participants did not return for blood sample collection at the end of the study (2 in ≥8% weight loss group, 7 in <8% weight loss group). Baseline characteristics of the 61 participants who completed the study, stratified by weight loss group, are reported in Table 1. There were no differences in sex distribution, age, initial weight, initial BMI, and body composition measurements between those who achieved ≥8% weight loss and those who did not.

### 3.2. Hormone and Cytokine Levels

The geometric means for each hormone and cytokine before and after weight loss for each weight loss group are presented in Table 2. In the ≥8% weight loss group, baseline to the 4-month levels decreased significantly for c-peptide (1,030 versus 891 pg/mL, *P* = 0.002), insulin (665 versus 541 pg/mL, *P* = 0.001), and leptin (0.83 versus 0.58 ng/mL/kg fat, *P* < 0.001). In the <8% weight loss group, baseline to the 4-month levels trended towards a decrease for MCP-1 (86.0 versus 72.96 pg/mL, *P* = 0.053). Compared to the ≥8% weight loss group, the <8% weight loss group had a significantly smaller change in leptin levels from baseline to month 4 (30% in ≥8% weight loss group versus 0% in <8% weight loss group, *P* = 0.012), a higher leptin level at month 4 (0.58 in ≥8% weight loss group versus 0.87 ng/mL/kg fat in <8% weight loss group, *P* = 0.048), and a tendency for a larger change in MCP-1 levels from baseline to month 4 (0% in ≥8% weight loss group versus 15% reduction in <8% weight loss group, *P* = 0.073). None of the analytes measured predicted ≥8% weight loss. 

## 4. Discussion

In this study with overweight and obese men and women (baseline BMI 27–40 kg/m^2^) placed on customized caloric restriction between 500 and 1000 kcal/day, we found that 75% of participants lost ≥8% of their starting body weights. Participants who lost ≥8% body weight had significantly lower c-peptide, insulin, and leptin levels after weight loss compared to baseline. Of the 12 hormones and cytokines measured, none predicted weight loss of ≥8%.

 Although those who achieved ≥8% weight loss had decreases in adipose signals after weight loss, these could have been consequences of weight loss rather than predictors. Decreased c-peptide, insulin, and leptin levels after weight loss have been documented in multiple studies [6–11]. We did not see a weight loss effect on secretion of fasting gastric inhibitory peptide (GIP).

Increases in fasting ghrelin levels have been observed in other populations of obese individuals in weight loss programs [8–10]. Weight loss effects on GLP-1 and PYY remain unclear with some studies finding no effect [12, 13] as we did, but others finding decreased fasting [14, 15] and postprandial levels [15, 16] after weight loss. The lack of change in the satiation signals we measured may be explained by two reasons: (1) satiation markers are more likely to be affected after a meal and (2) signals are generally stronger for hunger than satiation in a reduced obese state [17].

 Sumithran and colleagues published results from a meal replacement study on hormones involved in body weight regulation in participants achieving ≥10% weight loss [18]. Leptin and insulin levels decreased. Weight loss was also associated with reductions in PYY, amylin, and CCK and increases in ghrelin, PP, and postprandial GIP. They may have found these additional differences because their targeted weight loss was higher than ours (10% versus 8%, resp.). Their participants were on a very low-calorie diet, while ours were on a low-calorie diet. Very low-calorie diets may upregulate secretion of hunger signals and downregulate satiety signals as the energy deficit is more extreme than that of a low-calorie diet. As they excluded the participants who were unable to achieve ≥10% weight loss, no comparisons between successful and unsuccessful dieters were made.

 There was no profile of biomarkers related to adiposity, hunger, satiety, and inflammation that predicted weight loss success in our study. However, lower leptin and higher adiponectin before weight loss, as well as decreases in leptin and increases in adiponectin with weight loss, have predicted more weight loss in other programs with caloric-restriction diets [3, 5]. 

 Although leptin did not predict successful weight loss in our study, the changes observed in this hormone may promote weight regain over time implying more important roles during long-term weight loss maintenance. In studies employing short-term energy-restricted diets and following subjects long term for weight outcomes, lower baseline leptin predicted better weight loss maintenance [4], while weight regain has been associated with higher baseline leptin in women [6], larger decreases in leptin with weight loss [5], and lower baseline ghrelin in men [6]. 

In this cognitive-behavioral weight control program, behavioral factors at baseline or changes in behavior with weight loss may have outweighed the effects of physiological signals for determining successful weight loss. Broadly speaking, positive behavioral predictors of successful acute weight loss with diet or diet plus exercise include (1) positive self-perceptions (body image [19, 20], self-motivation [20], change in exercise self-efficacy [21], eating behavior self-efficacy [22], and prediction of success [20, 23]), (2) weight loss history (few or no prior weight loss attempts [19, 20]), and (3) treatment adherence (session attendance [21], treatment compliance [23]). Taken together, in diet-reduced individuals, behavioral factors may play a more significant role in acute weight loss success than physiological factors, but physiological factors may have larger effects on weight regulation during weight loss maintenance.

A strength of our study was that weight reduction was achieved with caloric restriction alone and was not confounded by increases in activity levels. Physical activity can alter markers of hunger and satiety [24–26]. We had high subject retention with nearly 94% (61/65) participants reaching the end of the weight loss intervention. This is in contrast with many other studies of similar duration and content whose attritions rates vary between 15% and 59% [27]. We were able to obtain blood samples from most participants in the <8% weight loss group even though they knew they would not continue on in the study. Finally, 75% of our participants were able to achieve weight loss success.

 Our study had some limitations. We obtained only fasting hormone and cytokine levels. Some of these biomarkers are more likely to change in a postprandial state. However, our aim was to identify markers that could be measured easily in clinical venues, which makes fasting samples more feasible than 2-hour postprandial or postglucose loads.

## 5. Conclusions

In our study of overweight and obese men and women placed on customized caloric restriction, there were no biomarkers able to predict successful weight loss. We observed significant changes in adiposity signals (leptin, insulin, and c-peptide) in the group successfully able to lose weight. Markers of satiation and inflammation did not significantly change within groups. Behavioral influences may override biological influences in individuals successfully able to lose weight. More research is needed to elucidate factors that predict ability to lose weight so that interventions can be targeted to optimize weight loss results.

## Figures and Tables

**Table 1:** Baseline participant characteristics.

Characteristic	Weight loss group		*P* value
	≥8% (*n* = 46)	<8% (*n* = 15)	
Female (%)	91.3	93.3	0.808
Age (years)	36.8 ± 6.9	35.0 ± 6.8	0.374
Weight (kg)	87.9 ± 12.6	89.7 ± 15.8	0.645
BMI (kg/m^2^)	31.6 ± 3.5	32.4 ± 4.3	0.476
Body composition^a^:			
Fat mass (kg)	34.7 ± 6.5	38.4 ± 10.5	0.113
Fat-free mass (kg)	49.2 ± 8.4	49.1 ± 6.4	0.960
Body fat (%)	40.3 ± 4.7	42.2 ± 5.6	0.191

BMI: body mass index.

^a^Mean ± SD.

**Table 2:** Hormone and cytokine levels before and after weight loss.

Subject characteristics and analytes^a^	≥8% WLG		<8% WLG	
	Baseline (*n* = 46)	4 months (*n* = 44)	Baseline (*n* = 15)	4 months (*n* = 8)
Weight (kg)	87.9 ± 12.6	78.2 ± 11.4	89.7 ± 15.8	88.4 ± 18.8^b^
BMI (kg/m^2^)	31.6 ± 3.5	28.27 ± 3.32	32.4 ± 4.3	31.51 ± 5.51^b^
Amylin (pg/mL)	38.3 (24.6–59.4)	41.0 (26.3–64.1)	24.4 (11.3–52.7)	21.8 (8.4–56.6)
C-Peptide (pg/mL)	1,030 (852–1,244)	891 (736–1,077)^c^	904 (649–1,260)	948 (663–1,356)
GIP (pg/mL)	93.8 (84.5–104.1)	89.8 (80.8–99.8)	83.3 (69.4–99.9)	90.2 (71.7–113.4)
Ghrelin (pg/mL)	28.2 (24.0–33.1)	32.6 (27.6–38.4)	23.8 (17.95–31.6)	28.2 (19.3–41.1)
GLP-1 (pg/mL)	7.90 (5.73–10.89)	7.50 (5.42–10.38)	4.83 (2.75–8.47)	4.46 (2.28–8.73)
IL-6 (pg/mL)	2.48 (1.91–3.24)	2.83 (2.16–3.70)	2.73 (1.72–4.33)	1.71 (0.93–3.13)
Insulin (pg/mL)	665 (573–772)	541 (465–628)^c^	532 (410–690)	461 (339–626)
Leptin (ng/mL/kg fat)^d^	0.83 (0.70–0.98)	0.58 (0.49–0.69)^c^	0.84 (0.62–1.13)	0.87 (0.61–1.23)^e,f^
MCP-1 (pg/mL)	81.5 (74.5–89.2)	81.6 (74.5–89.4)	86.0 (73.5–100.7)	72.96 (60.3–88.3)
PP (pg/mL)	81.3 (55.8–118.6)	64.8 (44.2–94.9)	57.7 (29.8–111.6)	44.4 (20.1–97.99)
PYY (pg/mL)	44.6 (33.1–60.1)	45.4 (33.5–61.5)	51.7 (30.6–87.2)	40.6 (21.0–78.4)
TNF-*α* (pg/mL)	3.76 (3.34–4.22)	3.86 (3.43–4.35)	3.72 (3.04–4.57)	3.24 (2.48–4.24)

WLG: weight loss group; GIP: gastric inhibitory peptide; GLP: glucagon-like peptide; IL: interleukin; MCP: monocyte chemotactic protein; PP: pancreatic polypeptide; PYY: peptide YY; TNF-*α*: tumor necrosis factor-alpha.

^a^Mean ± SD for subject characteristics and geometric mean (95% confidence interval) for analytes.

^b^ *P* < 0.001 for difference in change from baseline to 4 months between weight loss groups.

^c^ *P* ≤ 0.002 for ratio of baseline to 4-month measurements within weight loss group.

^d^Adjusted for fat mass.

^e^ *P* < 0.05 for difference between 4-month measurements between weight loss groups.

^f^ *P* < 0.05 for difference in change from baseline to 4 months between weight loss groups.
